# Supplementary material for: Causal interplay between lactose intolerance and gut microbiota: a combined bidirectional Mendelian randomization and in vivo validation study
Source: Front Nutr. 2026 Jun 1;13:1803337. doi: 10.3389/fnut.2026.1803337 (PMC13265576; doi:10.3389/fnut.2026.1803337)
Supplement: Supplementary file 1 [file Data_Sheet_1.zip › supplementary materials/Forward/leave one out cross validation/ebi-a-GCST90027702.finngen_R12_E4_LACTONAS.pdf]

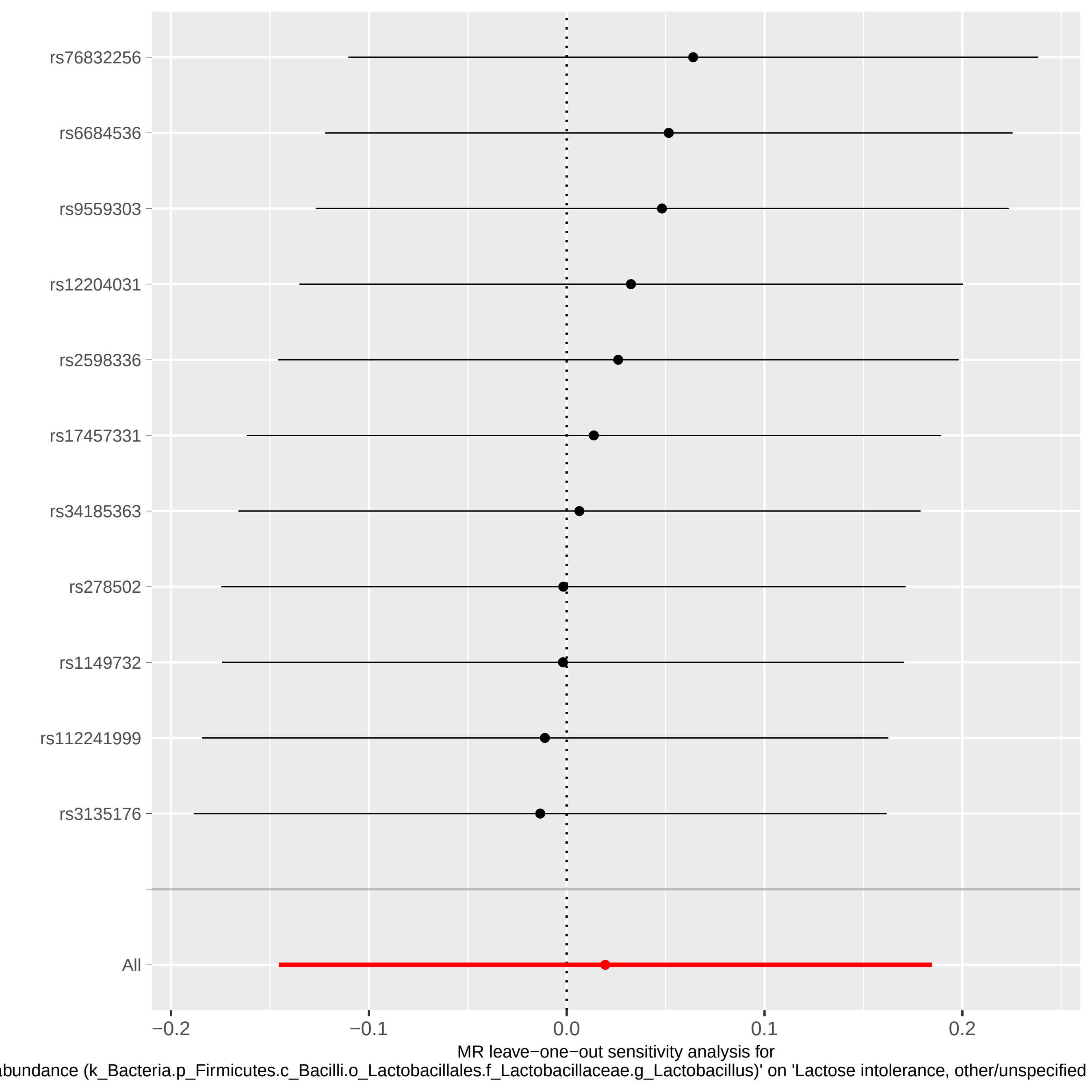
rs76832256
rs6684536
rs9559303
rs12204031
rs2598336
rs17457331
rs34185363
rs278502
rs1149732
rs112241999
rs3135176
All
−0.2
−0.1
0.0
0.1
0.2
MR leave−one−out sensitivity analysis for
abundance (k_Bacteria.p_Firmicutes.c_Bacilli.o_Lactobacillales.f_Lactobacillaceae.g_Lactobacillus)' on 'Lactose intolerance, other/unspecified
